# Gastrointestinal involvement in very early and established systemic sclerosis: insights from the SPRING-SIR national Italian registry

**DOI:** 10.1093/rheumatology/keaf457

**Published:** 2025-09-05

**Authors:** Francesco Bonomi, Cosimo Bruni, Silvia Peretti, Rossella De Angelis, Gianluigi Bajocchi, Dilia Giuggioli, Martina Orlandi, Giovanni Zanframundo, Rosario Foti, Giovanna Cuomo, Alarico Ariani, Edoardo Rosato, Gemma Lepri, Francesco Girelli, Valeria Riccieri, Elisabetta Zanatta, Silvia Laura Bosello, Ilaria Cavazzana, Francesca Ingegnoli, Maria De Santis, Fabio Cacciapaglia, Giuseppe Murdaca, Giuseppina Abignano, Pettiti Giorgio, Alessandra Della Rossa, Maurizio Caminiti, Anna Maria Iuliano, Giovanni Ciano, Lorenzo Beretta, Gianluca Bagnato, Ennio Lubrano, Ilenia De Andres, Luca Idolazzi, Marta Saracco, Cecilia Agnes, Corrado Campochiaro, Edoardo Cipolletta, Marco Fornaro, Federica Lumetti, Amelia Spinella, Luca Magnani, Giacomo De Luca, Veronica Codullo, Elisa Visalli, Carlo Iandoli, Antonietta Gigante, Greta Pellegrino, Erika Pigatto, Maria Grazia Lazzaroni, Enrico De Lorenzis, Gianna Mennillo, Marco Di Battista, Giuseppa Pagano-Mariano, Federica Furini, Licia Vultaggio, Simone Parisi, Clara Lisa Peroni, Gerolamo Bianchi, Enrico Fusaro, Gian Domenico Sebastiani, Marcello Govoni, Salvatore D’Angelo, Franco Cozzi, Franco Franceschini, Serena Guiducci, Lorenzo Dagna, Andrea Doria, Carlo Salvarani, Maria Antonietta D’Agostino, Florenzo Iannone, Clodoveo Ferri, Marco Matucci-Cerinic, Silvia Bellando Randone, SPRING-SIR (Systemic Sclerosis PRogression INvestiGation group of the Italian Society for Rheumatology), Francesco Bonomi, Cosimo Bruni, Silvia Peretti, Rossella De Angelis, Gianluigi Bajocchi, Dilia Giuggioli, Martina Orlandi, Giovanni Zanframundo, Rosario Foti, Giovanna Cuomo, Alarico Ariani, Edoardo Rosato, Gemma Lepri, Francesco Girelli, Valeria Riccieri, Elisabetta Zanatta, Silvia Laura Bosello, Ilaria Cavazzana, Francesca Ingegnoli, Maria De Santis, Fabio Cacciapaglia, Giuseppe Murdaca, Giuseppina Abignano, Pettiti Giorgio, Alessandra Della Rossa, Maurizio Caminiti, Anna Maria Iuliano, Giovanni Ciano, Lorenzo Beretta, Gianluca Bagnato, Ennio Lubrano, Ilenia De Andres, Luca Idolazzi, Marta Saracco, Cecilia Agnes, Corrado Campochiaro, Edoardo Cipolletta, Marco Fornaro, Federica Lumetti, Amelia Spinella, Luca Magnani, Giacomo De Luca, Veronica Codullo, Elisa Visalli, Carlo Iandoli, Antonietta Gigante, Greta Pellegrino, Erika Pigatto, Maria Grazia Lazzaroni, Enrico De Lorenzis, Gianna Mennillo, Marco Di Battista, Giuseppa Pagano-Mariano, Federica Furini, Licia Vultaggio, Simone Parisi, Clara Lisa Peroni, Gerolamo Bianchi, Enrico Fusaro, Gian Domenico Sebastiani, Marcello Govoni, Salvatore D’Angelo, Franco Cozzi, Franco Franceschini, Serena Guiducci, Lorenzo Dagna, Andrea Doria, Carlo Salvarani, Maria Antonietta D’Agostino, Florenzo Iannone, Clodoveo Ferri, Marco Matucci-Cerinic, Silvia Bellando Randone

**Affiliations:** Division of Rheumatology, Department of Experimental and Clinical Medicine, University of Florence, Florence, Italy; Department of Internal Medicine, University Hospital Careggi, Florence, Italy; Division of Rheumatology, Department of Experimental and Clinical Medicine, University of Florence, Florence, Italy; Department of Rheumatology, University Hospital Zurich, University of Zurich, Zurich, Switzerland; Division of Rheumatology, Department of Experimental and Clinical Medicine, University of Florence, Florence, Italy; Department of Internal Medicine, University Hospital Careggi, Florence, Italy; Rheumatology Unit, Department of Clinical and Molecular Sciences, Polytechnic University of Marche, Ancona, Italy; Rheumatology Unit, S. Maria Hospital-USL, IRCCS Institute, Reggio Emilia, Italy; Department of Medical and Surgical Sciences for Children and Adults, University Hospital of Modena and Reggio Emilia School of Medicine, Modena, Italy; Department of Medical and Surgical Sciences for Children and Adults, University Hospital of Modena and Reggio Emilia School of Medicine, Modena, Italy; Department of Internal Medicine and Therapeutics, Università di Pavia, Pavia, Italy; Division of Rheumatology, Fondazione IRCCS Policlinico San Matteo, Pavia, Italy; Rheumatology Unit, AOU Policlinico San Marco, Catania, Italy; Department of Precision Medicine, Univeristy of Campania—Luigi Vanvitelli University, Naples, Italy; Department of Medicine, Internal Medicine and Rheumatology, Azienda Ospedaliero Universitaria di Parma, Parma, Italy; Department of Translational and Precision Medicine, Sapienza University of Rome, Rome, Italy; Department of Internal Medicine, University Hospital Careggi, Florence, Italy; Department of Medicine, Rheumatology Unit, Ospedale GB Morgagni–L Pierantoni, Forlì, Italy; Department of Internal Medicine, Anesthesiology and Cardiovascular Sciences, Sapienza University of Rome, Rome, Italy; Department of Rheumatology, University of Padua, Padova, Italy; Rheumatology Division, Catholic University of the Sacred Heart, Fondazione Policlinico Universitario A. Gemelli—IRCCS, Rome, Italy; Rheumatology and Clinical Immunology, ASST Spedali Civili of Brescia, Brescia, Italy; Department of Clinical and Experimental Sciences, University of Brescia, Brescia, Italy; Clinica Reumatologica, Dipartimento di Reumatologia e Scienze Mediche, ASST Gaetano Pini-CTO, Dipartimento di Scienze Cliniche e di Comunità, Dipartimento di Eccellenza 2023-2027, Università degli Studi di Milano, Milan, Italy; Department of Biomedical Sciences, Humanitas University, Pieve Emanuele-Milan and Research Hospital, Milan, Italy; Rheumatology Unit, Department of Precision and Regenerative Medicine-Ionian Area, University of Bari “Aldo Moro”, Bari, Italy; Department of Medicine and Surgery, LUM ‘G. De Gennaro’, Reumatology Service, “F.Miulli” General Hospital, Casamassima, Bari, Italy; Department of Internal Medicine, University of Genoa, Genoa, Italy; Allergology and Clinical Immunology Unit, ospedale San Bartolomeo, Sarzana, Italy; Rheumatology Unit, Department of Health Science, University of Basilicata, San Carlo Hospital, Potenza, Italy; Rheumatology Unit ASO S., Croce e Carle Hospital, Cuneo, Italy; Department of Rheumatology, University of Pisa, Pisa, Italy; Departmental Rheumatology Unit, Grande Ospedale Metropolitano, Reggio Calabria, Italy; Rheumatology Unit, San Camillo–Forlanini Hospital, Rome, Italy; Local Health Department, Hospital of Ariano Irpino, Ariano Irpino, Italy; Referral Center for Systemic Autoimmune Diseases, Fondazione IRCCS Ca’ Granda, Ospedale Maggiore Policlinico di Milano, Milan, Italy; Department of Clinical and Experimental Medicine, University of Messina, Messina, Italy; Department of Rheumatology, University of Molise, Campobasso, Italy; Rheumatology Unit, Azienda Ospedaliera di Rilievo Nazionale ed Alta Specializzazione Garibaldi, Catania, Italy; Rheumatology Unit, Department of Medicine, University of Verona, Verona, Italy; Rheumatology Unit, Mauriziano-Umberto I Hospital, Turin, Italy; Division of Rehabilitation, Department of Medicine, Torino, ASL TO5, Carmagnola, TO, Italy; Unit of Immunology, Rheumatology, Allergy and Rare Diseases (UnIRAR), IRCCS San Raffaele Scientific Institute, and Vita-Salute San Raffaele University, Milan, Italy; Rheumatology Unit, Department of Clinical and Molecular Sciences, Polytechnic University of Marche, Ancona, Italy; Rheumatology Unit, Department of Precision and Regenerative Medicine-Ionian Area, University of Bari “Aldo Moro”, Bari, Italy; Department of Medical and Surgical Sciences for Children and Adults, University Hospital of Modena and Reggio Emilia School of Medicine, Modena, Italy; Department of Medical and Surgical Sciences for Children and Adults, University Hospital of Modena and Reggio Emilia School of Medicine, Modena, Italy; Rheumatology Unit, S. Maria Hospital-USL, IRCCS Institute, Reggio Emilia, Italy; Unit of Immunology, Rheumatology, Allergy and Rare Diseases (UnIRAR), IRCCS San Raffaele Scientific Institute, and Vita-Salute San Raffaele University, Milan, Italy; Department of Internal Medicine and Therapeutics, Università di Pavia, Pavia, Italy; Rheumatology Unit, AOU Policlinico San Marco, Catania, Italy; Department of Precision Medicine, Univeristy of Campania—Luigi Vanvitelli University, Naples, Italy; Department of Translational and Precision Medicine, Sapienza University of Rome, Rome, Italy; Department of Rheumatology, IRCCS Ospedale Galeazzi- Sant’Ambrogio, Milan, Italy; Dipartimento di Scienze Biomediche e Cliniche, Università degli Studi di Milano, Milan, Italy; Department of Medicine, Villa Salus Hospital, Mestre, Italy; Rheumatology and Clinical Immunology, ASST Spedali Civili of Brescia, Brescia, Italy; Department of Clinical and Experimental Sciences, University of Brescia, Brescia, Italy; Rheumatology Division, Catholic University of the Sacred Heart, Fondazione Policlinico Universitario A. Gemelli—IRCCS, Rome, Italy; Rheumatology Unit, Department of Health Science, University of Basilicata, San Carlo Hospital, Potenza, Italy; Department of Rheumatology, University of Pisa, Pisa, Italy; Departmental Rheumatology Unit, Grande Ospedale Metropolitano, Reggio Calabria, Italy; Rheumatology Unit, Department of Medical Sciences, University of Ferrara and Azienda Ospedaliera-Universitaria S. Anna, Ferrara, Italy; Rheumatology Unit, Department of Medical Sciences, University of Ferrara and Azienda Ospedaliera-Universitaria S. Anna, Ferrara, Italy; Rheumatology Unit, Azienda Ospedaliera Universitaria Città Della Salute e della Scienza di Torino, Turin, Italy; Rheumatology Unit, Azienda Ospedaliera Universitaria Città Della Salute e della Scienza di Torino, Turin, Italy; Rheumatology Unit, Department of Medical Specialities, Local Health Trust 3, Genoa, Italy; Rheumatology Unit, Azienda Ospedaliera Universitaria Città Della Salute e della Scienza di Torino, Turin, Italy; Rheumatology Unit, San Camillo–Forlanini Hospital, Rome, Italy; Rheumatology Unit, Department of Medical Sciences, University of Ferrara and Azienda Ospedaliera-Universitaria S. Anna, Ferrara, Italy; Rheumatology Unit, Department of Health Science, University of Basilicata, San Carlo Hospital, Potenza, Italy; Department of Medicine, Villa Salus Hospital, Mestre, Italy; Rheumatology and Clinical Immunology, ASST Spedali Civili of Brescia, Brescia, Italy; Department of Clinical and Experimental Sciences, University of Brescia, Brescia, Italy; Department of Internal Medicine, University Hospital Careggi, Florence, Italy; Unit of Immunology, Rheumatology, Allergy and Rare Diseases (UnIRAR), IRCCS San Raffaele Scientific Institute, and Vita-Salute San Raffaele University, Milan, Italy; Department of Rheumatology, University of Padua, Padova, Italy; Department of Medical and Surgical Sciences for Children and Adults, University Hospital of Modena and Reggio Emilia School of Medicine, Modena, Italy; Rheumatology Division, Catholic University of the Sacred Heart, Fondazione Policlinico Universitario A. Gemelli—IRCCS, Rome, Italy; Rheumatology Unit, Department of Precision and Regenerative Medicine-Ionian Area, University of Bari “Aldo Moro”, Bari, Italy; Department of Medical and Surgical Sciences for Children and Adults, University Hospital of Modena and Reggio Emilia School of Medicine, Modena, Italy; Rheumatology Clinic ‘Madonna dello Scoglio’ Crotonei, Crotone, Italy; Unit of Immunology, Rheumatology, Allergy and Rare Diseases (UnIRAR), IRCCS San Raffaele Scientific Institute, and Vita-Salute San Raffaele University, Milan, Italy; Division of Rheumatology, Department of Experimental and Clinical Medicine, University of Florence, Florence, Italy

**Keywords:** systemic sclerosis, VEDOSS, gastrointestinal involvement, oesophageal dysmotility, autoantibodies, disease progression

## Abstract

**Objectives:**

To describe the prevalence of gastrointestinal (GI) symptoms in SSc and Very Early Diagnosis of SSc (VEDOSS), identify clinical and serological features associated with GI involvement and explore a cranio-caudal pattern of symptom distribution, using data from the Italian SPRING-SIR registry.

**Methods:**

This cross-sectional analysis included patients fulfilling 2013 ACR/EULAR SSc or VEDOSS criteria. GI involvement was defined as symptoms in at least one GI tract segment and categorized as upper and lower. Associations between GI involvement and clinical variables were assessed using logistic and ordinal regression models, adjusting for demographics, disease characteristics and autoantibodies.

**Results:**

Among 1917 SSc patients, 56% had GI symptoms, associated with longer disease duration, dcSSc, interstitial lung disease (ILD), digital ulcers (DU), telangiectasias and tobacco exposure. Extensive GI involvement correlated with more severe disease. Ordinal regression identified female sex, dcSSc, ILD, DU, telangiectasias, tobacco exposure and anti-centromere antibodies as variables significantly associated with more extensive GI involvement. Disease duration did not show a significant association with GI symptom extent. Among 211 VEDOSS patients, 41.2% reported GI symptoms (mostly oesophageal), significantly associated with puffy fingers and dyspnoea. Among VEDOSS, puffy fingers and anti-centromere antibodies were independent predictors of presence of oesophageal symptoms.

**Conclusion:**

GI involvement in SSc is linked to more severe disease and longer disease duration. Disease duration resulted linked to the presence of GI symptoms rather than extent of GI involvement. Puffy fingers and anti-centromere antibodies may associate with presence of early oesophageal symptoms in VEDOSS.

## Graphical Abstract

**Figure:**
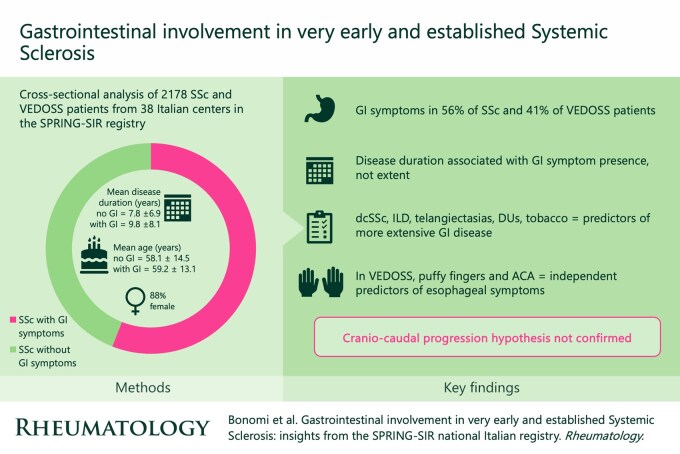

Rheumatology key messages

- Gastrointestinal symptoms are frequent in SSc and VEDOSS, correlating with severe systemic involvement.
- In SSc, disease duration predicts onset of gastrointestinal involvement, while vascular features correlate with extent.
- In VEDOSS, puffy fingers and anti-centromere antibodies associate with early oesophageal involvement.

## Introduction

Systemic sclerosis (SSc) is a rare, chronic and heterogeneous systemic autoimmune rheumatic disease characterized by the combination of vascular damage, immune dysregulation and fibrosis [1]. Clinically, SSc is a multisystem disease that can affect various organ systems, with significant morbidity associated with pulmonary, cardiovascular and gastrointestinal (GI) involvement [2]. GI complications are common in SSc, affecting up to 90% of patients at some point during the course of the disease [3]. GI complications vary widely, ranging from mild symptoms such as dysphagia, gastroesophageal reflux and bloating, to more severe manifestations like gastroparesis, paralytic ileus and intestinal pseudo-obstruction [4]. In SSc, GI involvement is not only a source of considerable discomfort but also contributes significantly to the overall disease burden, impacting patients’ quality of life, vitality and mental health, with established associations with anxiety and depression [5], and complicating their long-term management [6].

Despite the high prevalence, GI manifestations of SSc remain often under-recognized and undertreated, partly due to their heterogeneous nature and the reliance on subjective symptom-based assessments rather than specific diagnostic biomarkers [7–9]. Therefore, it is difficult for clinicians to accurately monitor disease progression and stratify patients based on their risk of severe GI complications. This results in suboptimal management with few targeted therapies available and a paucity of well-established treatment guidelines [10].

In the management of SSc GI involvement, the challenge is represented by the uncertainties that characterize the various domains. In fact, although fibrosis and vascular abnormalities are central to disease progression, the exact mechanisms leading to GI damage are not fully understood [11]. It is hypothesized that, over the course of SSc, GI involvement follows a cranio-caudal progression, starting from the oesophagus and advancing to the intestines [12]. Clinical observations and studies indicate that oesophageal dysmotility may precede other GI manifestations, such as small bowel and large intestine involvement, but this hypothesis still awaits to be proved. Additionally, the variability in symptom progression over time underlines the need for a personalized approach to patient care. Distinct trajectories of GI complications have been identified in SSc patients, suggesting that while some patients experience rapid progression, others maintain relatively stable GI function [13]. These findings, along with the identification of risk factors such as female sex, smoking and specific autoantibodies [14–17], have identified pulmonary fibrosis and extensive cutaneous involvement [15] as risk factors for more severe GI manifestations. Despite these associations, reliable biomarkers for predicting GI involvement remain elusive, highlighting the need for improved risk stratification strategies.

Identifying patients who are likely to develop severe GI complications and determining the optimal timing for screening and interventions is essential for improving outcomes [12]. Therefore, our study aims to address key gaps in understanding of GI involvement in SSc by describing the prevalence of GI symptoms in established SSc and Very Early Diagnosis of SSc (VEDOSS) populations using nationwide data from the SSc Progression INvestiGation (SPRING) registry of the Italian Society of Rheumatology (SIR). Moreover, our aim was to identify potential predictors of presence and extent of GI involvement, while also testing the hypothesis of a cranio-caudal pattern of progression of GI symptoms and to investigate whether disease duration may be associated with this pattern of progression, contributing to a more accurate profiling of patients based on their individual risk factors.

## Patients and methods

We conducted a cross-sectional analysis of baseline data from patients enrolled in the SPRING-SIR registry. The SPRING-SIR registry is a prospective, multicentre cohort study that has consecutively enrolled SSc-spectrum cases since 2015, up to June 2022. This strategic, non-profit initiative is led by the Italian Society for Rheumatology (SIR) and involves 38 centres across Italy [18]. Patients were not selected based on disease duration or severity. Thus, the cohort includes both early and established cases. Longitudinal follow-up data were not analysed in this study due to high numbers of loss to follow-ups and short follow-up duration. The study protocol was approved by the Ethics Committees of all participating centres following the authorization of the coordinating centre (reference number OSS 15.010, AOU Careggi, Florence). All participants provided written informed consent before taking part in the study. Data collection and management were performed using Research Electronic Data Capture (REDCap), a secure web-based platform designed for supporting clinical research data.

From the SPRING-SIR registry, we included both patients diagnosed with SSc who met the 2013 American College of Rheumatology/European League Against Rheumatism (ACR/EULAR) [19] classification criteria for SSc and VEDOSS patients, defined as patients with Raynaud’s phenomenon (RP) and at least one other VEDOSS criterion (ANA, SSc antibodies, puffy fingers or scleroderma pattern on nailfold videocapillaroscopy), but who did not meet the 2013 ACR/EULAR SSc classification criteria. Patients with missing information on GI symptoms were excluded.

In the SPRING-SIR registry, GI symptoms are differentiated into oesophageal (defined as patient-reported presence of reflux or dysphagia), gastric (defined as patient-reported early satiety and vomiting) and intestinal tracts (defined as patient-reported diarrhoea, bloating or constipation). ‘GI involvement’ was defined as the presence of symptoms in at least one of these regions, while the extent of involvement was classified into two categories: ‘upper GI involvement’, which referred to presence of esophageal or gastric symptoms (but not intestinal), and ‘lower GI involvement’, which included intestinal symptoms regardless of the presence of upper GI symptoms. In addition, patients were arbitrarily stratified in 5 groups according to disease duration, defined as the time from the onset of the first non-Raynaud’s phenomenon sign or symptoms (≤3 years, >3 and ≤6 years, >6 and ≤9 years, >9 and ≤12 years, >12 years). Definitions of other clinical variables (e.g. ILD, scleroderma renal crisis, myositis, PAH) followed the criteria adopted in the SPRING-SIR registry, based on clinical signs, laboratory tests, imaging (e.g. HRCT for ILD) and functional assessments (e.g. PFTs, echocardiography), as detailed in the original registry publication [20].

Descriptive analyses were performed separately for the SSc and VEDOSS cohorts, using χ^2^ or Student’s *t* test, as appropriate, to compare patients with and without GI involvement, as well as groups with different extent of GI involvement. Fisher’s test was performed, when appropriate.

Multivariable logistic regression analysis was performed to identify predictors of presence of GI involvement and to evaluate which red flags were associated to a more extensive GI disease in the SSc cohort. In the VEDOSS cohort, due to sample size issues, logistic regression analysis was performed only to identify predictors of presence of GI involvement, with a sensitivity analysis focused on esophageal symptoms. To investigate the cranio-caudal progression hypothesis, which assumes a sequential progression from no GI involvement to isolated upper GI involvement, and finally to combined upper and lower GI involvement, we performed an ordinal regression analysis. This statistical method assumes a linear, unidirectional progression between these ordered categories, without reversal to less severe states, and identifies risk factors associated with progression between consecutive steps. The model presumes that the risk conferred by each predictor remains consistent across all transitions.

All regression models considered factors such as anti-centromere antibody positivity [16], female sex [16], tobacco exposure [16], telangiectasias [21, 22] and digital ulcers [23] as potential predictors, as these have been previously linked to the presence or progression of GI involvement in other publications. Additionally, we explored the potential correlation with interstitial lung disease (ILD), as a connection between gastroesophageal reflux and ILD is widely recognized [24–28]. We also included diffuse cutaneous SSc (dcSSc), based on clinical expertise and evidence from literature [29–32] suggesting that these patients tend to exhibit more extensive GI involvement. Lastly, age and disease duration were included in the models, as it is commonly believed that GI involvement follows a cranio-caudal progression, with its extent worsening over time, although literature data are limited [11, 12]. All statistical analyses were performed using SPSS version 29.0, and a *P*-value of <0.05 was considered statistically significant.

## Results

### Prevalence and characteristics of patients with GI involvement

Out of 2178 enrolled patients, 1917 met the inclusion criteria for definite SSc cohort and 211 for the VEDOSS cohort. Fifty patients were excluded due to missing information regarding GI symptoms. The demographic and clinical characteristics of SSc patients with and without GI symptoms are detailed in Supplementary Table S1. Overall, 56% of the SSc patients reported GI symptoms at baseline visit. Patients with GI symptoms exhibited a longer disease duration (9.84 *vs* 7.75 years, *P* < 0.001) as well as a higher prevalence of dcSSc (23.8% *vs* 13.2%, *P* < 0.001) and ILD (43.5% *vs* 31%, *P* < 0.001), in line with poorer lung function values. Alongside these clinical features, patients with GI involvement had significantly higher frequencies of telangiectasias (68.0% *vs* 49.1%, *P* < 0.001), digital ulcers (25.4% *vs* 15.6%, *P* < 0.001) and digital pitting scars (54.2% *vs* 36%, *P* < 0.001) and were more frequently treated with vasoactive-vasodilating therapies.

When patients were stratified by the extent of GI involvement—from those without any GI symptoms to those with isolated upper GI manifestations, and further to those with both upper and lower GI symptoms—similar findings emerged (Table 1). Patients with combined upper and lower GI involvement had the longest disease duration (11.6 ± 9.5 years) and a markedly higher prevalence of dcSSc (28%, *P* < 0.001) and sclerodactyly (76.8%, *P* < 0.001), indicative of more aggressive fibrotic phenotype compared with cases with isolated upper or no GI involvement. Similarly, vascular abnormalities were also progressively more pronounced in groups with higher extent of GI involvement (late nailfold videocapillaroscopy pattern seen in 19.8% patients without GI symptoms, 28.1% with upper GI symptoms and 33.8% with late GI symptoms, *P* < 0.001). ILD was also increasingly more present alongside a higher GI symptom burden: with a prevalence of 47.1% in patients with both upper and lower GI involvement compared with 42.3% in those with isolated upper GI symptoms and 31% in patients without GI symptoms (*P* < 0.001).

**Table 1.** Comparison of demographical data and symptoms in SSc patients with different extent of GI involvement at time of enrolment in the SPRING-SIR registry

No. of patients, 1917	GI extent				
	No GI symptoms	Upper GI symptoms	Upper and lower GI symptoms	Missing (n)	*P* value
	844 (45.8)	704 (38.2)	293 (15.9)	76	
Female sex, *n* (%)	742 (88.1)	619 (88.1)	265 (91.1)	81	0.345
Age, mean (SD)	58.1 (14.1)	59.3 (13.2)	59.5 (12.9)	78	0.147
Disease duration, mean (SD)	8.8 (7.7)	10.8 (8.6)	11.6 (9.5)	264	**<0.001**
Tobacco exposure, *n* (%)	223 (29.3)	218 (35.3)	98 (37.1)	274	**0.015**
Diffuse cutaneous involvement, *n* (%)	111 (13.2)	159 (22.6)	82 (28.0)	76	**<0.001**
Sclerodactyly, *n* (%)	506 (60.1)	539 (76.6)	225 (76.8)	78	**<0.001**
Puffy fingers, *n* (%)	442 (52.4)	358 (50.9)	142 (48.6)	78	0.531
Digital pitting scars, *n* (%)	303 (36.0)	377 (53.6)	174 (59.4)	78	**<0.001**
Digital ulcers, *n* (%)	132 (15.6)	164 (23.3)	96 (32.8)	76	**<0.001**
Telangiectasias, *n* (%)	414 (49.1)	455 (64.6)	217 (74.6)	78	**<0.001**
Calcinosis, *n* (%)	56 (6.7)	88 (12.5)	65 (22.2)	79	**<0.001**
Joints contractures, *n* (%)	70 (8.3)	95 (13.5)	75 (25.6)	77	**<0.001**
Tendon friction rubs, *n* (%)	40 (4.7)	62 (8.8)	51 (17.5)	77	**<0.001**
Arthritis, *n* (%)	76 (9.1)	89 (12.7)	42 (14.5)	88	**0.014**
Anti-topoisomerase I, *n* (%)	286 (34.3)	239 (34.3)	100 (35.1)	100	0.970
Anti-RNApolimerase III, *n* (%)	12 (1.8)	10 (1.8)	6 (2.7)	461	0.685
Anti-centromere, *n* (%)	264 (34.1)	196 (29.9)	93 (34.2)	215	0.190
Nailfold videocapillaroscopy pattern late, *n* (%)	151 (19.8)	177 (28.1)	91 (33.8)	257	**<0.001**
Interstitial lung disease, *n* (%)	262 (31.0)	298 (42.3)	138 (47.1)	76	**<0.001**
Dyspnoea, *n* (%)	218 (26.0)	305 (43.3)	173 (59.5)	83	**<0.001**
DLCO, mean (SD)	72.6 (19.9)	66.2 (19.9)	64.4 (21.5)	618	**<0.001**

Statistically significant results are shown in bold.

### GI involvement increases with disease duration

**Figure 1.**
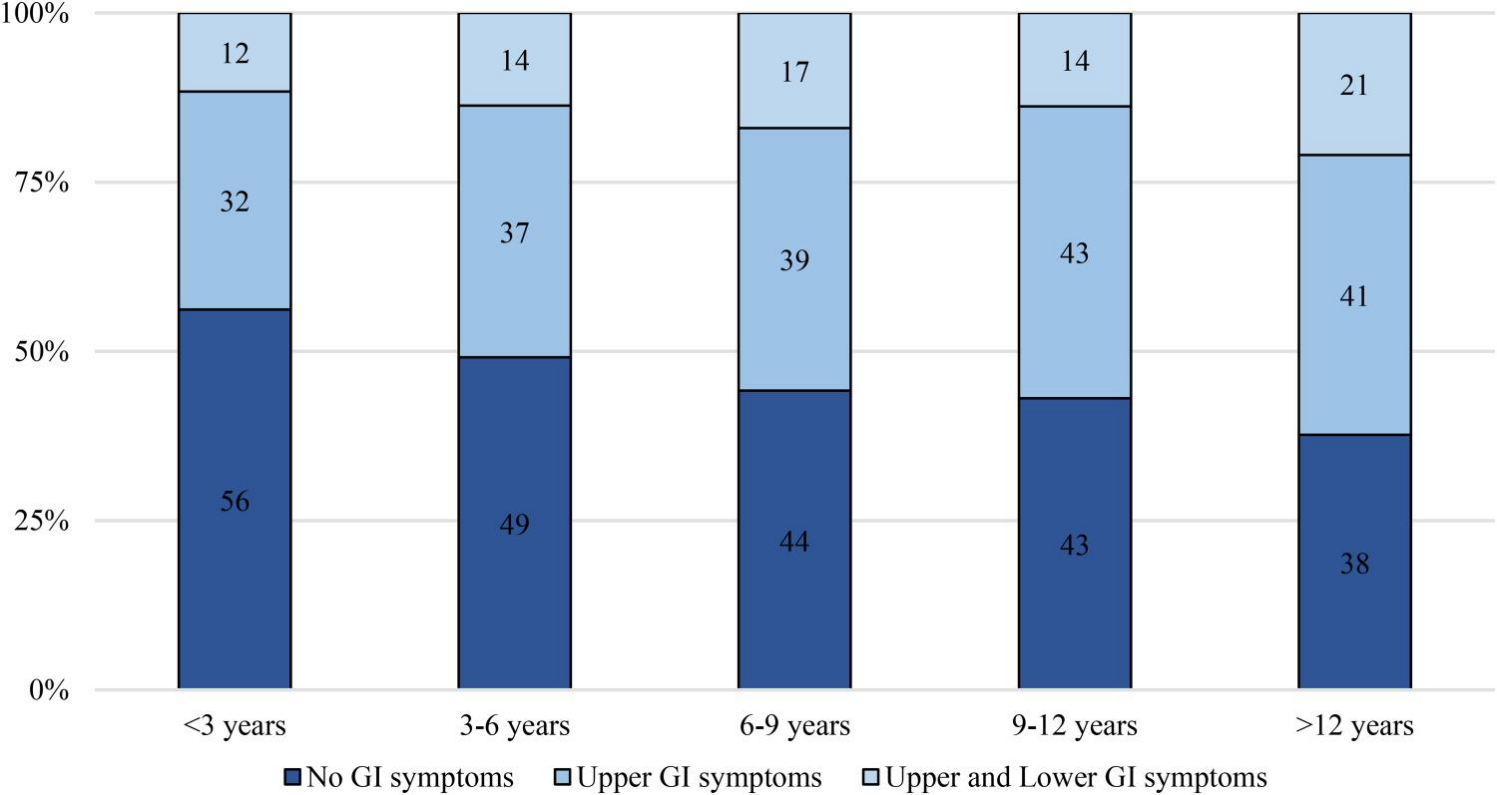
Stratification of GI extent according to disease duration, representing the time from the first non-Raynaud’s phenomenon sign or symptom

Looking at the prevalence of GI involvement across groups with different disease durations at the time of enrolment in the registry, we observed that the frequency of patients without GI symptoms decreased progressively—from 56.2% in those with a disease duration of <3 years to 37.7% in patients with >12 years—while the prevalence of GI symptoms correspondingly increased from 43.8% to 62.3% (*P* < 0.001). When patients were stratified by the extent of GI involvement, both the prevalence of isolated upper GI symptoms and combined upper and lower GI symptoms increased in those with longer disease duration, with a significant difference between the three groups (*P* < 0.001). Specifically, patients with isolated upper GI symptoms increased from 32.2% in those with <3 years of disease to 41.3% in those with >12 years, while the prevalence of combined upper and lower GI symptoms rose from 11.6% to 21% (Fig. 1).

### Predicting presence and extent of GI involvement

**Figure 2.**
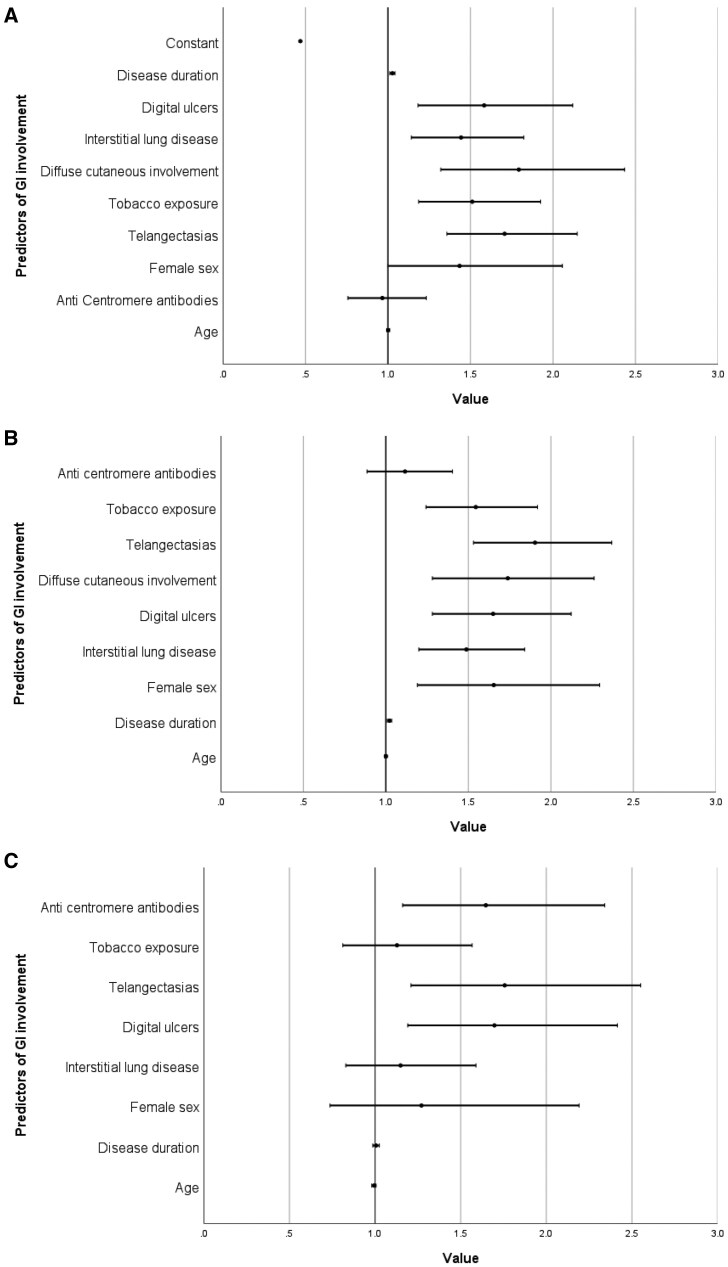
Regression models of determinants of presence, extent and distribution of GI involvement in SSc. Forest plots showing the multivariable logistic (**A**, **C**) and ordinal (**B**) regression models evaluating factors associated with (**A**) the presence of GI involvement, (**B**) the extent of GI involvement (increase in number of affected GI segments) and (**C**) the pattern of GI involvement (upper GI *vs* combined upper-and-lower GI involvement) in patients with SSc. The plot displays odds ratios (ORs) and 95% confidence intervals (CIs) for each covariate included in the model. All variables included in the models were categorical, except for age and disease duration (years)

Multivariable logistic regression identified several independent parameters associated with GI involvement (Fig. 2A). Specifically, the presence of telangiectasias (OR 1.707, 95% CI 1.357–2.147, *P* < 0.001), tobacco exposure (OR 1.511, 95% CI 1.186–1.925, *P* < 0.001), dcSSc (OR 1.793, 95% CI 1.320–2.434, *P* < 0.001), ILD (OR 1.443, 95% CI 1.142–1.823, *P* = 0.002), digital ulcers (OR 1.583, 95% CI 1.182–2.120, *P* = 0.002) and longer disease duration (OR 1.027, 95% CI 1.011–1.043, *P* = 0.001) were all significantly associated with GI involvement.

Ordinal regression further confirmed that prolonged disease duration (OR 1.022, 95% CI 1.009–1.035, *P* < 0.001) along with ILD (OR 1.488, 95% CI 1.200–1.841, *P* < 0.001), dcSSc (OR 1.739, 95% CI 1.282–2.262, *P* < 0.001), digital ulcers (OR 1.650, 95% CI 1.282–2.123, *P* < 0.001), telangiectasias (OR 1.904, 95% CI 1.531–2.369, *P* < 0.001), female sex (OR 1.654, 95% CI 1.191–2.296, *P* = 0.003) and tobacco exposure (OR 1.545, 95% CI 1.243–1.919, *P* < 0.001), were independently associated with a greater extent of GI involvement (Fig. 2B). Specifically, these findings imply that each of these factors significantly increased the likelihood of progression from no involvement to isolated upper GI involvement, and ultimately to combined upper and lower GI involvement. Interestingly, we additionally performed an ordinal regression analysis using a four-level model: no GI symptoms, isolated esophageal involvement, combined esophageal and gastric symptoms, and full involvement of oesophagus, stomach and intestines (presented in Supplementary Table S2). This stepwise model confirmed the same set of independent predictors (longer disease duration, ILD, dcSSc, digital ulcers, telangiectasias, female sex and tobacco exposure), reinforcing the consistency of our findings across different classifications of GI symptom extent.

Moreover, when analysing risk factor associated with more severe disease extent (isolated upper GI symptoms *vs* combined upper and lower involvement), digital ulcers (OR 1.697, 95% CI 1.191–2.415, *P* = 0.003), telangiectasias (OR 1.757, 95% CI 1.211–2.551, *P* = 0.003) and anti-centromere antibodies (OR 1.647, 95% CI 1.161–2.342, *P* = 0.005) emerged as significant predictors, while disease duration did not reach significance in this specific analysis (OR 1.006, 95% CI 0.987–1.025, *P* = 0.560) (Fig. 2C).

### GI involvement in VEDOSS

Of the 211 enrolled VEDOSS patients, 87 (41.2%) reported GI symptoms at baseline: 75 (35.5%) esophageal, 23 (10.9%) gastric and 26 (12.3%) intestinal symptoms, respectively. The demographic and clinical characteristics of patients with and without GI symptoms are detailed in Table 2. Although no statistically significant difference was observed in terms of age, sex, antibody positivity and time from Raynaud’s phenomenon onset, VEDOSS patients with GI symptoms presented significantly more frequently puffy fingers (29.9% *vs* 15.3%, *P* = 0.016), dyspnoea (27.6% *vs* 6.5%, *P* < 0.001) and sicca symptoms (40.2% *vs* 18.5%, *P* < 0.001). No difference was found in terms of prevalence of telangiectasias, ILD and concomitant medications. In both groups, history of digital ulcers was negative.

**Table 2.** Comparison of demographic and clinical data in VEDOSS with and without GI involvement

Parameter	No GI symptoms	Presence of GI symptoms	Missing (n)	*P* value
	**124 (58.8)**	**87 (41.2)**	**0**	
Age, mean (SD)	52.1 (15.8)	53.7 (13.4)	0	0.428
Time from Raynaud’s phenomenon, mean, (SD)	9.19 (9.8)	10.57 (10.1)	0	0.318
Time from Raynaud’s phenomenon onset, *n* (%)				
<5 years	51 (41.1)	30 (34.5)		
5–10 years	32 (25.8)	22 (25.3)		
>10 years	41 (33.1)	35 (40.2)	0	0.516
Tobacco exposure, *n* (%)	30 (27.8)	34 (41.5)	21	0.063
Female sex, *n* (%)	112 (91.1)	92 (94.3)	1	0.441
ANA, *n* (%)	122 (98.4)	79 (96.3)	5	0.389
Anti-topoisomerase I, *n* (%)	14 (11.3)	12 (14.6)	5	0.524
Anti-RNApolimerase III, *n* (%)	2 (1.8)	1 (1.4)	28	>0.999
Anti-centromere, *n* (%)	42 (36.2)	35 (43.8)	15	0.301
Nailfold videocapillaroscopy pattern				
Early, *n* (%)	51 (44.3)	31 (37.8)		
Active, *n* (%)	22 (19.1)	17 (20.7)		
Late, *n* (%)	3 (2.6)	0 (0.0)	14	0.391
Sicca syndrome, *n* (%)	23 (18.5)	35 (40.2)	0	**<0.001**
Dyspnoea, *n* (%)	8 (6.5)	24 (27.6)	1	**<0.001**
Puffy fingers, *n* (%)	19 (15.3)	26 (29.9)	0	**0.016**
Telangiectasias, *n* (%)	7 (5.6)	11 (12.6)	0	0.131
Calcinosis, *n* (%)	2 (1.6)	1 (1.1)	0	>0.999
Arthritis, *n* (%)	8 (6.5)	7 (8.1)	1	0.786
Interstitial lung disease, *n* (%)	1 (0.8)	4 (4.6)	0	0.162

Fisher test was used when fewer than five cases were present. Statistically significant results are shown in bold.

Logistic regression identified puffy fingers as the only statistically significant predictor of presence of any GI involvement among VEDOSS patients (OR 2.174, 95% CI 1.082–4.371, *P* = 0.029). When specifically focusing on esophageal symptoms, as the most prevalent type of GI involvement in this subgroup, puffy fingers were confirmed as an independent predictor (OR 2.806, 95% CI 1.346–5.849, *P* = 0.006), along with anti-centromere antibodies (OR 2.075, 95% CI 1.104–3.899, *P* = 0.023).

## Discussion

Our study provides comprehensive insights into the intricate relationship between GI involvement and other clinical manifestations of definite SSc and VEDOSS patients, highlighting possible key predictors associated with the presence of GI symptoms. The findings highlight the substantial burden of GI symptoms in SSc (more than half of the patients in our cohort) and reveal significant associations with systemic disease severity, pulmonary and cutaneous manifestations, and increased treatment need.

Patients with GI symptoms had a longer disease duration, consistent with previous studies showing that GI involvement tends to progress alongside the SSc course [16, 33]. Disease duration was identified as an independent predictor of both upper and lower GI symptoms, with the highest prevalence observed in patients with a disease duration exceeding 12 years. Interestingly, while disease duration independently predicted the presence of both upper and lower GI symptoms, it was not associated with the progression from upper to lower GI involvement. This challenges the commonly held assumption that GI manifestations in SSc follow a cranio-caudal (upper-to-lower) progression, suggesting instead that upper and lower GI involvement may develop independently rather than representing sequential stages of a single disease process.

Our findings deepen the knowledge in GI tract involvement in SSc and complements the findings of recent literature. Our cross-sectional registry-based data provide insights into the anatomical distribution of GI symptoms and their relationship with disease duration and systemic manifestations at the time of first clinical consultation. In their elegant analysis, Perin *et al.* used longitudinally collected Medsger GI severity scores to capture the temporal dynamics of GI burden in terms of severity [13]. Interestingly, patients in the ‘early progressive’ and ‘early severe’ GI clusters had longer disease duration at baseline compared with the ‘late progressive’ and ‘stable’ clusters, the latter including over 80% of their large SSc cohort. However, since the Medsger score reflects symptom severity rather than anatomical extent, patients classified as ‘stable’ may still experience progression in terms of anatomical disease extent (e.g. from esophageal to lower GI involvement) without a marked change in severity scores. Likewise, an increase in severity of upper GI—esophageal disease may not be associated to a further disease extent to the lower, intestinal GI tract.

These methodological differences in the two studies highlight that the two approaches are not directly comparable, but rather complementary. Taken together, these perspectives reinforce the importance of identifying patients in the early phases of disease, even when symptoms are mild or subclinical, to better characterize the natural history of GI involvement and start timely therapeutic strategies aimed at preventing irreversible damage.

Our data also confirm a robust association between GI involvement and severe pulmonary manifestations, such as ILD and reduced pulmonary function (DLCO and FVC). This relationship was further supported by the independent association between ILD and GI involvement observed in the logistic regression analysis, highlighting the interplay between these organ systems in SSc. One possible explanation for these findings lies in the hypothesis that persistent microaspirations [24] caused by reflux may contribute to progressive interstitial lung damage. While the causal relationship remains controversial, existing literature aligns with our results [25–27, 34], showing an association between severe esophageal dysfunction, such as absent peristalsis, and greater extent of pulmonary involvement. Additionally, it has been shown that the severity of reflux symptoms correlates with ILD progression over 2 years, further reinforcing the link between GI and pulmonary involvement in SSc [28].

Cutaneous manifestations also emerged as a clinical parameter which significantly correlates with GI involvement, in particular in dcSSc patients exhibiting a higher prevalence of GI symptoms. This association likely reflects the underlying pathophysiological mechanisms, such as widespread vascular dysfunction and fibrosis, which may contribute to both skin and GI tract involvement. Additionally, specific markers of severe skin and microvascular involvement, including digital ulcers, telangiectasias and digital pitting scars, were significantly more common in patients with GI symptoms, suggesting that these features could serve as ‘red flags’ for clinicians in identifying patients at higher risk for GI complications.

Furthermore, active tobacco exposure emerged as a significant predictor of GI symptoms, consistent with prior findings [35], highlighting the need for smoking cessation programs as part of disease management strategies.

Therapeutically, patients with GI involvement frequently received biologic DMARDs, endothelin receptor antagonists, PDE5 inhibitors and prostanoids, reflecting the higher prevalence of systemic complications, such as diffuse ILD and vascular involvement, in this subgroup rather than a direct link to GI involvement itself. However, GI involvement plays a crucial role in therapeutic decision-making, as the potential GI side effects of therapies can significantly impact patient preferences and treatment adherence. Recent studies underscore the importance of shared decision-making in optimizing treatment strategies for SSc, integrating patient preferences regarding potential adverse events, such as GI symptoms, into therapeutic planning [36, 37]. Future research should explore how targeted therapies, including immunomodulators and vascular agents, can address both systemic and GI disease manifestations while aligning with patient needs and preferences [38]. Additionally, a protective role of calcium channel blockers has been suggested in reducing the severity of GI symptoms [16], while a potential benefit from immunosuppressive treatment has been suggested on GI manifestations observing better UCLA GIT 2.0 scores in the cohort treated with immunosuppressants [39].

Finally, the association between autoantibodies and GI involvement in SSc remains inconsistent, also after our analysis. For instance, anti-centromere antibodies were linked to an increased risk of severity in GI involvement [16], whereas anti-topoisomerase I antibodies were associated with late progression of GI symptoms [13], suggesting that vascular dysfunction might play a key role in the progression of GI involvement. Although anti-centromere antibodies were not associated with a clear progression pattern [13], our data suggest that they might have a role in the onset of GI involvement. This hypothesis was further supported by our observation that anti-centromere antibodies were found to be predictors of esophageal symptoms in VEDOSS patients.

Consistent with this an early esophageal and anorectal dysfunction has been observed in VEDOSS patients [40], detecting functional abnormalities even among asymptomatic individuals. Specifically, although only 49% and 14% of patients reported esophageal and anorectal symptoms, respectively, esophageal dysfunction was observed in 25% of asymptomatic cases, and nearly all asymptomatic patients showed anorectal abnormalities [40]. Our findings reinforce these observations, showing a high prevalence of GI symptoms in VEDOSS patients, particularly esophageal, which often precedes other systemic SSc manifestations. This is particularly interesting as a recent machine learning analysis of the EUSTAR-VEDOSS registry has shown that the presence of esophageal symptoms significantly increases the predictive accuracy for progression to definite SSc [41]. Patients with Raynaud’s phenomenon, positive ANA and esophageal symptoms had a 55% progression rate over 5 years, compared with 47% in those without esophageal involvement. The inclusion of esophageal symptoms alongside SSc-specific autoantibodies identified individuals with a significantly shorter time-to-progression, suggesting that esophageal dysfunction may serve as an additional ‘red flag’ in risk stratification models. Furthermore, puffy fingers, a hallmark feature in the VEDOSS criteria, have been shown in previous studies as a critical 'red flag’ in patients with Raynaud’s phenomenon, signalling an increased risk of transitioning to SSc [42, 43]. Our findings support the notion that puffy fingers are not merely a clinical sign but also a critical marker of underlying disease progression, particularly when associated with GI symptoms. Together, these findings highlight the pathogenic potential of ACA and puffy fingers as early indicators of GI disease in SSc, and the importance of systematic early screening and longitudinal assessment of GI function in patients at risk.

Our study has some limitations: the first, its retrospective and cross-sectional design inherently limits the ability to establish causal relationships between risk factors and GI involvement and to evaluate symptom progression over time, which is not possible in the current setting due to the limited number of follow-up visits and of patients developing new GI events. In fact, a prospective longitudinal study would be needed to show the progression from upper to lower GI during the disease course. Additionally, GI involvement was solely assessed based on patient-reported symptoms: this could have potentially led to an under- or over-estimation of the actual GI prevalence, considering that GI involvement can often be asymptomatic and given also the clinical challenge of differentiating SSc- and non-SSc-related causes of GI symptoms. Also, the coding of GI symptoms in the registry was based on predefined categories, without the possibility of reliably determining their underlying pathogenesis; symptoms were assigned to the most typically involved anatomical region, in line with approaches used in other registries [44]. This approach, while ensuring uniformity in data collection, may have introduced a systematic misclassification bias, particularly for nonspecific symptoms such as bloating, which can originate from different segments of the GI tract. Furthermore, the coding used allowed only for the identification of the involved gastrointestinal tract, without enabling the assessment of symptom severity. Moreover, the SPRING registry did not include detailed information on GI-specific treatments, limiting insights into the therapeutic impact. Finally, we acknowledge the observational nature of the registry, including highly heterogeneous, prevalent population of SSc patients which might have contributed to a certain selection/survivor bias, as well as the limited ethnic diversity in our cohort (>98% Caucasian), which may partly impair the generalizability of our data to other geographical contexts.

## Conclusions

Our data highlight that a more severe disease phenotype in SSc is strongly associated with both the presence and extent of GI involvement. Independent risk factors for GI symptoms include disease duration, dcSSc, ILD, DUs and tobacco exposure. While disease duration is significantly linked to the presence of GI symptoms, our data could not indirectly associate this parameter with the progression from upper to lower GI involvement, suggesting a role more in the initiation rather than the evolution of GI complications. Our findings also confirm that in the VEDOSS cohort, GI symptoms are more prevalent than other SSc-related organ involvements and manifestations, with esophageal symptoms being particularly common. We identified puffy fingers and anti-centromere antibodies as significant predictors of GI involvement in VEDOSS patient, especially esophageal symptoms, suggesting their possible role as ‘red flags’ for progression to definite SSc. These insights emphasize the importance of early detection, personalized management and risk stratification to alleviate the burden of GI disease and improve patient outcomes over time. Further research is essential to better understand the mechanisms driving GI manifestations and refine screening and treatment strategies accordingly.

## Supplementary Material

keaf457_Supplementary_Data

## Data Availability

Data are available for research purposes upon request.
