# Supplementary material for: Gastrointestinal involvement in very early and established systemic sclerosis: insights from the SPRING-SIR national Italian registry
Source: Rheumatology (Oxford). 2025 Sep 5;65(1):keaf457. doi: 10.1093/rheumatology/keaf457 (PMC12862390; doi:10.1093/rheumatology/keaf457)
Supplement: keaf457_Supplementary_Data [file keaf457_supplementary_data.docx]

**Supplementary Table S1.** Demographic and clinical characteristics of SSc patients with and without GI symptoms

| **N° of patients 1917** | **GI involvement** | |  |  |
| --- | --- | --- | --- | --- |
|  | **No**  **GI symptoms** | **Presence of GI symptoms** | **Missing**  **(n)** | p value |
|  | **844 (44.0)** | **1073 (56.0)** | 0 |  |
| **Female sex, n (%)** | 742 (88.1) | 954 (89.2) | 5 | 0.513 |
| **Age, mean (SD)** | 58.1 (14.5) | 59.2 (13.1) | 2 | 0.081 |
| **Disease duration, mean (SD)** | 7.8 (6.9) | 9.8 (8.1) | 188 | **<0.001** |
| **Tobacco exposure, n, (%)** | 223 (29.3) | 349 (36.6) | 201 | **0.002** |
| **Cutaneous involvement extent**  **Limited, n (%)**  **Diffuse, n (%)** | 583 (69.1)  111 (13.2) | 691 (64.4)  255 (23.8) | 0 | **<0.001** |
| **Sclerodactyly, n, (%)** | 506 (60.1) | 807 (75.2) | 2 | **<0.001** |
| **Puffy fingers, n (%)** | 442 (54.2) | 543 (50.7) | 2 | 0.490 |
| **Digital pitting scars, n (%)** | 303 (36.0) | 581 (54.2) | 3 | **<0.001** |
| **Digital ulcers, n (%)** | 132 (15.6) | 273 (25.4) | 0 | **<0.001** |
| **Telangectasia, n, (%)** | 414 (49.1) | 728 (68.0) | 2 | **<0.001** |
| **Calcinosis n, (%)** | 56 (6.7) | 64 (15.3) | 3 | **<0.001** |
| **Joint contractures, n, (%)** | 70 (8.3) | 174 (16.2) | 1 | **<0.001** |
| **Tendon friction rubs n, (%)** | 40 (4.7) | 118 (11.0) | 1 | **<0.001** |
| **Arthritis, n, (%)** | 76 (9.1) | 136 (12.7) | 12 | **0.012** |
| **Anti topoisomerase I, n, (%)** | 286 (34.3) | 361 (34.1) | 24 | 0.961 |
| **Anti RNApolimerase III n, (%)** | 12 (1.8) | 16 (1.9) | 397 | 1 |
| **Anti Centromere, n, (%)** | 264 (34.1) | 312 (31.3) | 146 | 0.220 |
| **Nailfold videocapillaroscopy**  **Early n, (%)**  **Active n, (%)**  **Late n, (%)** | 191 (25.1)  369 (48.5)  151 (19.8) | 187 (19.3)  449 (46.2)  284 (25.9) | 185 | **<0.001** |
| **Interstitial lung disease, n, (%)** | 262 (31.0) | 467 (43.5) | 0 | **<0.001** |
| **Dyspnea, n, (%)** | 218 (26.0) | 506 (47.2) | 7 | **<0.001** |
| **DLCO, mean (SD)** | 72.6 (18.9) | 66.3 (20.6) | 542 | **<0.001** |
| **FVC, mean (SD)** | 104.1 (22.3) | 99.2 (22.5) | 478 | **<0.001** |
| **Disease duration n, (%)**  **<3 years**  **3-6 years**  **6-9 years**  **9-12 years**  **>12 years** | 211 (28.6)  165 (22.4)  103 (14.0)  85 (11.5)  173 (23.5) | 199 (20.1)  192 (19.4)  148 (14.9)  127 (12.8)  326 (32.9) | 188 | **<0.001** |

Statistically significant results are shown in bold.

**Supplementary Table S2.** Ordinal regression analysis using a four-level model of GI involvement: (1) no GI symptoms, (2) isolated esophageal symptoms, (3) combined esophageal and gastric symptoms, and (4) full involvement of esophagus, stomach, and intestines. The table reports odds ratios (OR), 95% confidence intervals (CI), and p-values for each tested variable.

| Tested variables | OR | 95%CI | | p-value |
| --- | --- | --- | --- | --- |
| Age | 1.005 | 0.996 | 1.013 | 0.271 |
| Disease duration | 1.019 | 1.005 | 1.033 | **0.006** |
| Female sex | 1.725 | 1.224 | 2.432 | **0.002** |
| Interstitial lung disease | 1,563 | 1,248 | 1,957 | **<0.001** |
| Digital ulcers | 1,894 | 1,451 | 2,475 | **<0.001** |
| Diffuse cutaneous SSc | 1,786 | 1,355 | 2,353 | **<0.001** |
| Telangectasias | 1,701 | 1,351 | 2,141 | **<0.001** |
| Anti-centromere antibody | 1,623 | 0,735 | 1,200 | 0.616 |
| Tobacco exposure | 1,527 | 1,214 | 1,919 | **<0.001** |
| Parallel lines assumption test |  |  |  | 0.065 |

Statistically significant results are shown in bold.
